# Image Patch-Based Net Water Uptake and Radiomics Models Predict Malignant Cerebral Edema After Ischemic Stroke

**DOI:** 10.3389/fneur.2020.609747

**Published:** 2020-12-23

**Authors:** Bowen Fu, Shouliang Qi, Lin Tao, Haibin Xu, Yan Kang, Yudong Yao, Benqiang Yang, Yang Duan, Huisheng Chen

**Affiliations:** ^1^College of Medicine and Biological Information Engineering, Northeastern University, Shenyang, China; ^2^Key Laboratory of Intelligent Computing in Medical Image, Ministry of Education, Northeastern University, Shenyang, China; ^3^Department of Neurology, General Hospital of Northern Theater Command, Shenyang, China; ^4^College of Health Science and Environment Engineering, Shenzhen Technology University, Shenzhen, China; ^5^Department of Electrical and Computer Engineering, Stevens Institute of Technology, Hoboken, NJ, United States; ^6^Department of Radiology, General Hospital of Northern Theater Command, Shenyang, China

**Keywords:** malignant cerebral edema, predictive model, radiomics, CT image, ischemic stroke, net water uptake

## Abstract

Malignant cerebral edema (MCE) after an ischemic stroke results in a poor outcome or death. Early prediction of MCE helps to identify subjects that could benefit from a surgical decompressive craniectomy. Net water uptake (NWU) in an ischemic lesion is a predictor of MCE; however, CT perfusion and lesion segmentation are required. This paper proposes a new Image Patch-based Net Water Uptake (IP-NWU) procedure that only uses non-enhanced admission CT and does not need lesion segmentation. IP-NWU is calculated by comparing the density of ischemic and contralateral normal patches selected from the middle cerebral artery (MCA) area using standard reference images. We also compared IP-NWU with the Segmented Region-based NWU (SR-NWU) procedure in which segmented ischemic regions from follow-up CT images are overlaid onto admission images. Furthermore, IP-NWU and its combination with imaging features are used to construct predictive models of MCE with a radiomics approach. In total, 116 patients with an MCA infarction (39 with MCE and 77 without MCE) were included in the study. IP-NWU was significantly higher for patients with MCE than those without MCE (*p* < 0.05). IP-NWU can predict MCE with an AUC of 0.86. There was no significant difference between IP-NWU and SR-NWU, nor between their predictive efficacy for MCE. The inter-reader and interoperation agreement of IP-NWU was exceptional according to the Intraclass Correlation Coefficient (ICC) analysis (inter-reader: ICC = 0.92; interoperation: ICC = 0.95). By combining IP-NWU with imaging features through a random forest classifier, the radiomics model achieved the highest AUC (0.96). In summary, IP-NWU and radiomics models that combine IP-NWU with imaging features can precisely predict MCE using only admission non-enhanced CT images scanned within 24 h from onset.

## Introduction

Stroke is the leading cause of death and disability, resulting in 5.9 million deaths and 102 million disability-adjusted life-years worldwide (1). Ischemic stroke accounts for about 85% of the total incidence (2). A focal occlusion at the middle cerebral artery (MCA) leads to large hemispheric infarctions in some patients since the MCA supplies a large amount of blood to the brain.

Progressive cerebral edema usually results in a space-occupying infarct. The edema increases both brain volume and intracranial pressure. In the first 1–3 days after the onset of stroke, an abrupt neurological decline associated with displacement of midline brain structures may occur in ~10% of the patients with ischemic stroke of the MCA (3–5). These tissue shifts and subsequent brain herniation make the mortality rate increase to nearly 80% and thus are termed malignant cerebral edema (MCE) or malignant MCA infarction (6, 7). MCA can be relieved by decompressive craniectomy performed within 48 h of stroke onset or before herniation (3, 8).

Early and precise prediction of MCE can help identify the patients who could potentially benefit from a surgical decompressive craniectomy. Moreover, it can also help clinicians prepare for possible deterioration and communicate with patients and their family members about the goals of care (5). Compared with MRI, CT is the most favorable imaging modality for the prediction of MCE due to its fast acquisition and widespread availability. For example, Minnerup et al. (6) proposed the use of CT-based cerebrospinal fluid (CSF) volume as a predictor of MCE. The ischemic lesion volume must be measured manually from the admission perfusion CT images (cerebral blood volume, CBV). The clot burden score and collateral score measured from CT angiography have also been considered as predictors of MCE (9). Ong et al. developed the Enhanced Detection of Edema in Malignant Anterior Circulation Stroke (EDEMA) to predict the risk of lethal malignant edema; it includes two CT imaging variables at 24 h after the midline shift and basal cistern effacement (10). The EDEMA score showed a higher positive predictive value (93%) than the baseline image markers, such as the Alberta Stroke Program Early CT score (ASPECTS) or hyperdense vessel sign. Cheng et al. added the National Institute of Health Stroke Score (NIHSS) into EDEMA and validated it using a dataset of Chinese patients (11). Two recent meta-analysis studies summarized additional potential predictors (12, 13).

Net water uptake (NWU) measured by non-enhanced CT is useful for predicting malignant infarctions. A CBV map driven from CT perfusion (CTP) can be used to precisely locate the early ischemic infarct core and a non-enhanced CT is applied to quantitatively measure density changes. The NWU is calculated using the formula 1-D_Ischemic_/D_Normal_, where D_Ischemic_ (HU) is the density of the ischemic core with hypoattenuation and D_Normal_ is the density of the area of the contralateral normal tissue (14–16).

Radiomics aims to extract high-dimensional and quantitative features from medical images that can be used to build predictive models with machine learning methods to support clinical decisions (17, 18). Radiomics has played an important role in the study of many diseases such as cancers (19, 20). For stroke management, radiomics have been used to predict recanalization in ischemic stroke and hematoma expansion (21, 22). However, no study on predicting MCE by radiomics has been reported.

Regarding NWU and the prediction of MCE, most previous studies required multimodal CT images including CTP or CTA. Some dedicated software packages involve tedious semiautomatic or even manual segmentation. Hence, we propose a new way of calculating NWU that uses only non-enhanced admission CT and does not require CTP, CTA, or segmentation of the ischemic core. We hypothesize that in patients with ischemic stroke due to MCA occlusion, a non-enhanced admission CT can be used to predict MCE by calculating NWU through pre-defined image patches on the affected and non-affected MCA areas. Moreover, combining NWU with clinical and imaging features enables the construction of radiomics models that can predict MCE at an early stage after an ischemic MCA stroke.

## Materials and Methods

### Participants and the Dataset

This retrospective single-center study was approved by the Medical Ethics Committee of the General Hospital of Northern Theater Command and no informed consent was required by the committee. The selection of patients was carried out in accordance with inclusion and exclusion criteria. The inclusion criteria for this study were the following: (1) patients who were diagnosed with an MCA infarction with the occlusion at the MCA M1 segment; (2) patients that had both non-enhanced CT images within 24 h on admission and non-enhanced CT images after 24 h as the follow-up scan; (3) demographic information was available from the time of the stroke onset to the CT scans, including NIHSS score, the use of interventional thrombectomy (IT), the use of bone flap surgery, and the outcome (death for stroke or not); and (4) the development of an MCE was known. Using these criteria, we selected 125 patients from archive data on patients who were admitted to the General Hospital of Northern Theater Command between April 2017 and December 2018. Nine patients were further excluded due to the poor quality of admission CT images at 24 h. Finally, a total of 116 patients were included in the study.

We declared patients to have an MCE if they had infarcts with a mass effect during the follow-up non-contrast CT after admission, had clinically experienced a cerebral hernia due to the mass effect of edema, received bone flap surgery, or died due to the mass effect. This definition is the same as that given by Broocks et al. (16).

CT images were acquired with a Discovery CT750 HD scanner (GE Healthcare, Milwaukee, WI, USA) with a tube voltage of 120 kVp, x-ray tube current of 300 mA, the protocol of Axial Head, a slice thickness of 5.0 mm, 20 mm spacing between slices, a matrix of 512 × 512, and voxel spacing of 0.449/0.449 mm. The CT image data is available upon request after approval from the General Hospital of Northern Theater Command, China.

### Net Water Uptake Calculated by Image Patches

**Figure 1:**
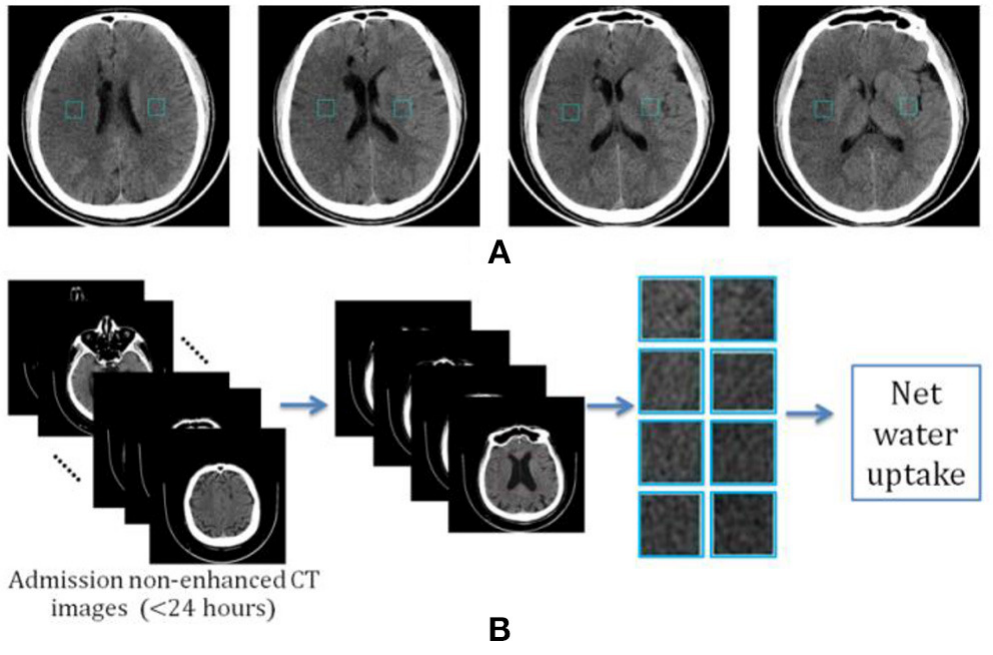
Determination of image patch-based net water uptake (IP-NWU). **(A)** The standard reference images with patches of 30 × 30; **(B)** The procedure for calculating IP-NWU.

Given the fact that an early hypoattenuated infarct (lesion core) is often not visible or that it can be difficult to precisely locate in non-enhanced CT images, we propose a new way of calculating net water uptake using CT patches determined using the standard reference images. After reviewing all the images, two experienced neuroradiologists selected four slices as the standard reference images and marked two mirrored patches of 30 × 30 voxels from the right and left MCA areas at each slice, as shown in Figure 1.

The criteria for determining the reference images included: (1) that patches should be located in the upper temporal lobe, the lateral parietal lobe, or the border area of the frontal, temporal, and parietal lobes; (2) the patches should avoid old lesions; (3) that patches should be located in the infarct area if there is an obvious infarct area; and, (4) the regions with CSF should be avoided to eliminate its effect on NWU. Subsequently, blinded to any clinical information, two other neuroradiologists independently located four pairs of patches from the images of each patient using these reference images and following the criteria mentioned above simultaneously.

Among each pair of patches, the example with hypoattenuation was considered to be ischemic and became density (HU) of D_Ischemic_; the other was the normal patch with the density of D_Normal_. Image patch-based net water uptake (IP-NWU) was calculated with the formula:

(1) $$\begin{array}{r} \text{IP}-\text{NWU}=1-\frac{{\text{D}}_{\text{Ischemic}}}{{\text{D}}_{\text{Normal}}}. \end{array}$$

### Net Water Uptake Calculated by Segmented Regions

**Figure 2:**
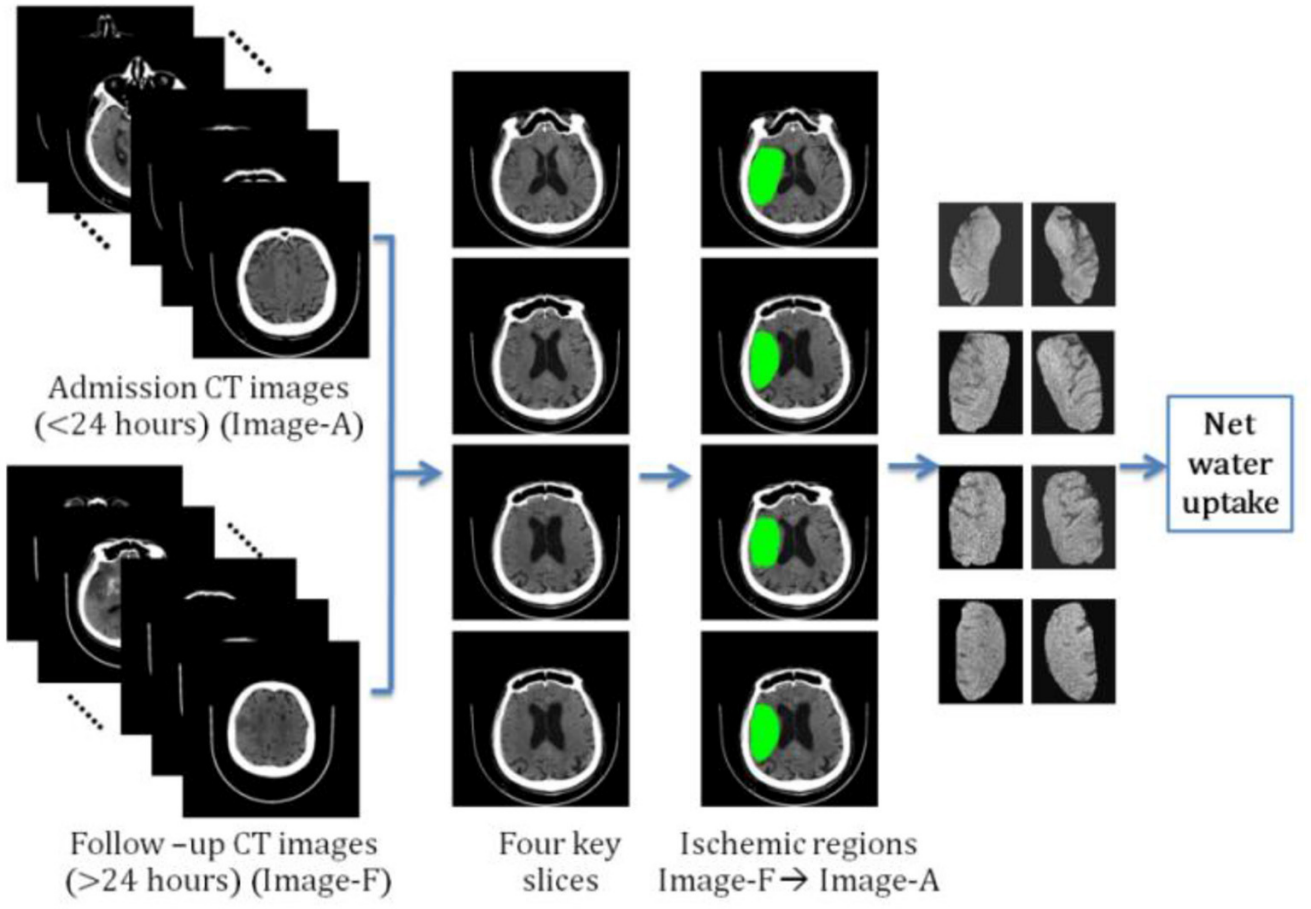
The procedure for calculating segmented region-based net water uptake (SR-NWU).

We determined another way of calculating NWU by manually segmenting the ischemic regions. The result was named the segmented region-based NWU (SR-NWU). As shown in Figure 2, first, both the admission CT images (<24 h; Image-A) and the follow-up CT images (>24 h; Image-F) were aligned and normalized to MNI-152 space by linear affine transformation with 12 degrees of freedom. Second, four slices were selected from Image-F using standard reference images and the ischemic regions were manually segmented. Finally, these regions were overlaid onto Image-A to calculate D_Ischemic_ from the CT intensity in Image-A, and D_Normal_ was determined from the mirrored region.

### Histogram Based Imaging Features

To fully utilize the information contained in the selected patches, we also calculated the voxel-wised IP-NWU maps ($\frac{{\text{D}}_{\text{Ischemic}}}{{\text{D}}_{\text{Normal}}}$, four 30 × 30 matrices with elements ranging from 0 to 1.0). Based on the four maps, the discrete histogram function can be depicted as

(2) $$\begin{array}{r} h\left(r_{n}\right)=Y_{n} \end{array}$$

where *r*_*n*_ is IP-NWU of the *n-*th grade, *Y*_*n*_ is the number of voxels with IP-NWU of *r*_*n*_, *n*=1, 2, .., N. Here N was set at 8.

For univariate data *Y*_1_, *Y*_2_, ..., *Y*_N_, five parameters could be calculated: (1) standard deviation (*s*); (2) slope; (3) entropy; (4) skewness (*g*); and (5) kurtosis. The slope was defined as the gradient between the minimum and maximum points among the vector of *Y*_1_, *Y*_2_, ..., *Y*_N_. Entropy was defined as

(3) $$\begin{array}{r} H=\overset{N}{\underset{n=1}{\sum }}p_{n}\log p_{n} \end{array}$$

where *p*_*n*_ is the ratio of the number of voxels with IP-NWU of *r*_*n*_ to the total number of voxels. *H* indicates the average amount of information in the image. The skewness was defined as

(4) $$\begin{array}{r} g=\frac{\sum_{i=1}^{N}{\left(Y_{i}-\bar{Y}\right)}^{3}/N}{s^{3}} \end{array}$$

where *s* is the standard deviation and $\bar{Y}$ is the mean. The skewness was near zero for the symmetric data, negative for the data skewed left, and positive for the data skewed right. The kurtosis is given as

(5) $$\begin{array}{r} k=\frac{\sum_{i=1}^{N}{\left(Y_{i}-\bar{Y}\right)}^{4}/N}{s^{4}}-3 \end{array}$$

Hence, *k* is zero for the standard normal distribution; it is positive for a “heavy-tailed” distribution and negative for a “light-tailed” distribution.

In total, 13 image features including *Y*_1_, *Y*_2_, ..., *Y*_8_, and the five parameters defined above were employed to construct the radiomics models for predicting MCE. The calculation of 13 radiomics features was done using the Python code written by our group.

### Machine Learning Algorithms

Three machine learning algorithms including support vector machine (SVM), logistic regression (LR), and random forest (RF) were employed as the classifier to predict MCE using IP-NWU, 13 image features, and three clinical features (age, gender, and NIHSS score).

For a training dataset *D* = {(_*x*_1_, *y*1_), (_*x*_2_, *y*2_), …, (_*x*_*m*_, *ym*_)}, *y*_*i*_ϵ{−1, +1}, the SVM algorithm draws each entity (_*x*_*i*_, *yi*_) in the dataset as a point in n-dimensional space (*n* is the number of features) and each feature is treated as a specific coordinate. The classification is carried out by finding a hyperplane (ω, *b*) that maximizes the margin between two categories (23–25). The learned parameters ω and *b* can be determined by solving the following equations.

(6) $$\begin{array}{r} \underset{\omega ,b}{\min}\frac{1}{2}\|\omega {\|}^{2} \end{array}$$

(7) $$\begin{array}{r} s.t.\;y_{i}\left(\omega^{T}x_{i}\right)\geq 1,\;i=1,\;2,\;\ldots ,\;m. \end{array}$$

LR is a kind of classic supervised learning method and it models the log odds (or logit) by linearly combining the independent variables (26).

(8) $$\begin{array}{r} \ln\left(\frac{y}{\left(1-y\right)}\right)=\omega^{T}x+b \end{array}$$

For a dataset ${\left\{\left({x_{i},y}_{i}\right)\right\}}_{i=1}^{m}$, *y*_*i*_ϵ{0, 1}, LR estimates ω and *b* by maximizing the log-likelihood

(9) $$\begin{array}{r} l\left(\omega ,b\right)=\overset{m}{\underset{i=1}{\sum }}\ln\;p\left(y_{i}|x_{i};\omega ,b;\right) \end{array}$$

where

(10) $$\begin{array}{r} p\left(y=1|x\right)=\frac{e^{w^{T}x+b}}{1+e^{w^{T}x+b}} \end{array}$$

(11) $$\begin{array}{r} p\left(y=0|x\right)=\frac{1}{1+e^{w^{T}x+b}} \end{array}$$

RF is a parallel-style ensemble learning method that uses a decision tree as the base learner and bagging as the ensemble strategy (27, 28). Each bootstrap sample generated through bagging with *m* observations was used to train one decision tree and a final consensus estimate was obtained by combining all individual bootstrap estimates. A subset *p* of *n* features was selected randomly for the partition of each node of the tree, which effectively reduced the similarity of trees generated from different bootstrap samples (29). One can refer to the specific literature on machine learning for more details about SVM, LR, and RF (30).

All three machine learning algorithms were implemented with Scikit-learn (an open source machine learning library) with default settings. Regarding the RF classifier, there was hyperparameter tuning and cross validation. Specifically, the optimal values of two hyperparameters of random_state and n_estimators were determined with a grid search: for random_state, a range of 2–16 with a step of 2 was used, and for n_estimators, a range of 100–1,000 with a step of 100 was used. The final optimal parameters were random_state = 10 and n_estimators = 100.

### Statistics and Performance Evaluation of Predictive **Models**

The inter-reader agreement for IP-NWU was evaluated with the intraclass correlation coefficient (ICC). If the ICC is larger than 0.75, then the reliability of the method for calculating NWU is good. The Bland-Altman statistical method was applied to assess the agreement between two methods of calculating NWU. For IP-NWU, the inter-reader and interoperation agreement were also assessed with the Bland-Altman method. A *p*-value of <0.05 was considered to indicate a significant difference.

The performances of various predictive models were evaluated with leave-one-out cross validation (LOOCV). It was implemented with Scikit-learn. In LOOCV, for a dataset with **m** samples, only one sample was left for the test and the others were used for training a model. This process was conducted for **m** times. LOOCV has been shown to almost estimate the generalizability of machine learning models impartially (31).

The receiver operating characteristic curve (ROC), the area under the ROC curve (AUC), the confusion matrix, accuracy (ACC), sensitivity (SEN), specificity (SPC), F1-score, positive predictive value (PPV), and negative predictive value (PPV) were calculated and compared. DeLong's method was used to evaluate whether there was a significant difference between two AUCs of ROC curves (32). Matthews correlation coefficient (MCC) was also used to evaluate and compare the performance of binary classifiers since it considers all fields of the confusion matrix (33).

## Results

### Clinical Characteristics

Among all 116 patients, 39 patients (13 female, 33.3%) were observed with MCE and 77 patients (32 female, 41.6%) were without MCE. There was no significant difference in NIHSS scores between groups with MCE and without MCE [median, 12; interquartile range (IQR), 7 vs. median, 15; IQR, 4.5; *p* = 0.2288]. The time from stroke onset to the first CT scan was longer in patients with MCE (mean 8.28 h vs. mean 5.32 h; *p* < 0.05). However, the time from stroke onset to the second CT scan was equivalent for the two groups (mean 35.42 h vs. mean 36.90 h; *p* > 0.05). The rate of IT was only 15.52% (18 of 116, 4 in the MCE group, 14 in the non-MCE group). Among the 18 patients treated with IT, 11 had achieved complete perfusion and 7 had achieved partial perfusion according to the Thrombolysis in Cerebral Infarction perfusion (TICI) scale (34). The four patients in the MCE group achieved complete perfusion after IT treatment. All the above characteristics of patients are listed in Table 1.

**Table 1:** Characteristics of patients with a middle cerebral artery ischemic stroke.

**Characteristic**	**Patient with MCE, *n* = 39**	**Patients without MCE, *n* = 77**	***p***
Demographics			–
Age, year, mean (SD)	64.23 (±11.39)	65.79 (±12.05)	0.5993^a^
Women, No. [%]	13 [33.3%]	32 [41.6%]	0.3059^b^
NIHSS score			0.2288^a^
Median (IQR)	12 (±7)	15 (±4.5)	–
Mean (SD)	11 (±6.83)	15 (±2.94)	–
With IT, No. [%]	4 [21.1%]	14 [18.2%]	0.2654^b^
Time from stroke onset to the first CT scan (within 24 h), hour, mean (SD)	8.28 (±6.53)	5.32 (±4.11)	0.0038^a^
Time from stroke onset to the second CT scan (beyond 24 h), hour, mean (SD)	35.42 (±8.83)	36.90 (±10.70)	0.4600^a^

*MCE, Malignant cerebral edema; CT, Computed tomography; IT, Interventional thrombectomy; NIHSS, National Institutes of Health Stroke Scale; SD, Standard deviation; IQR, Interquartile range*.

a *Two-sample t-test*;

b *Chi-squared test*.

### IP-NWU and Its Value With Time

**Figure 3:**
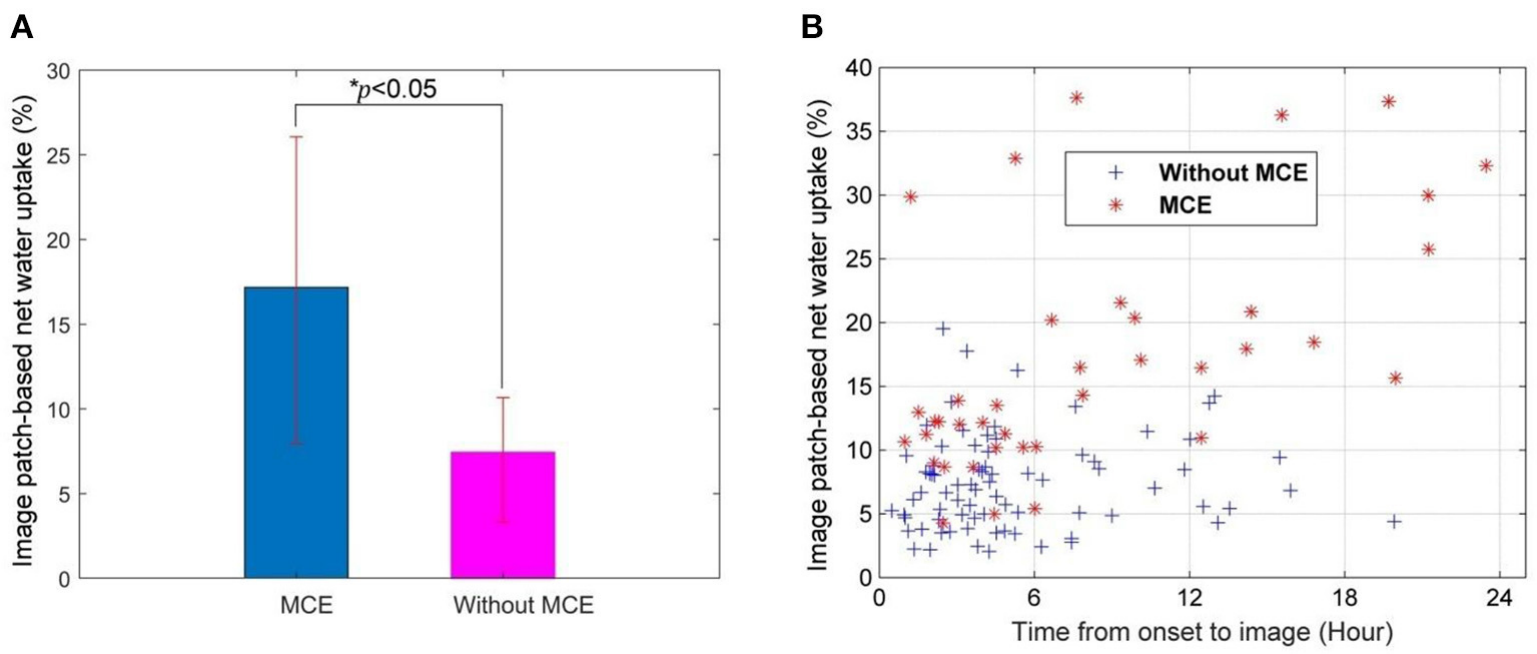
Image patch-based net water uptake (IP-NWU) and its relationship with time. **(A)** IP-NWU comparison between patients with MCE and without MCE; **(B)** The relationship between IP-NWU and time from stroke onset to imaging.

IP-NWU in patients who developed MCE was significantly higher than that in those without MCE (*p* < 0.05; Figure 3A). The average of IP-NWU in these two groups was 18.2 and 8.5%. These values were very close to those given by a previous study (18.0 and 7.0%) where semiautomatic segmentation of core lesions was done with the aid of CT perfusion images (16).

IP-NWU in both groups increased from the time of onset to imaging (Figure 3B). However, the edema rate for the group with MCE was larger than that of the group without MCE.

### IP-NWU as a Predictor of MCE and the Influence of IT and Time on Predictions

**Figure 4:**
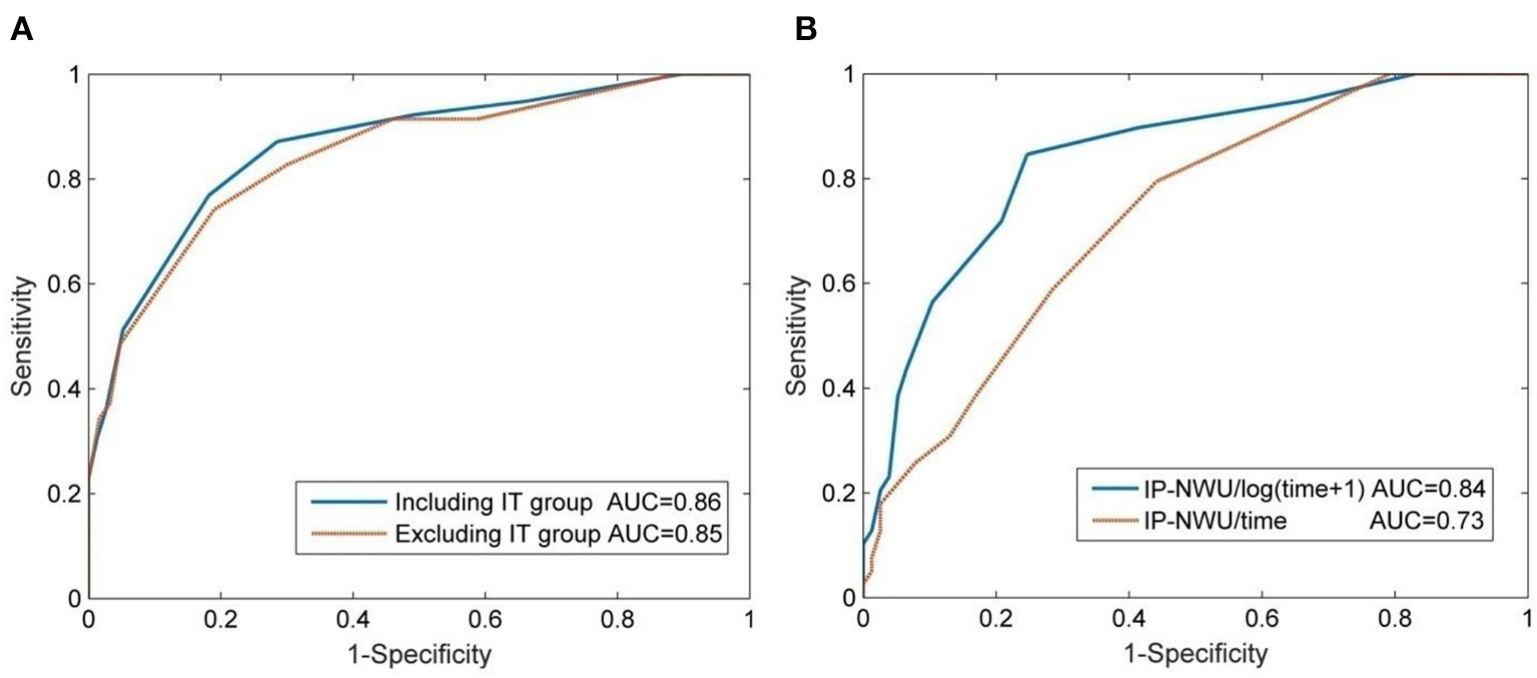
Prediction of malignant cerebral edema (MCE) by IP-NWU and its confounders. **(A)** ROC curve of prediction of MCE by IP-NWU for groups including patients who underwent interventional thrombectomy (IT) and excluding patients who underwent IT; **(B)** ROC curve by IP-NWU/time and by IP-NWU/log(time+1).

The optimal cut-off value of IP-NWU for discriminating between the patients with MCE and without MCE was 12.25%. Using this cut-off value, the predictive model could achieve a SEN of 0.64, an SPE of 0.91, and an ACC of 0.82. Univariate ROC curve analysis of IP-NWU resulted in an AUC of 0.86 (Figure 4A).

As for ROC curve predictions of MCE by IP-NWU, there was no significant difference between groups including and excluding patients who underwent IT (DeLong test, *p* > 0.05; MCC, 0.58 vs. 0.55; Figure 4A). This result demonstrated that interventional thrombectomy does not influence the prediction of MCE when using IP-NWU. These findings are in accordance with previous observations (16).

The predictive power of IP-NWU/time and IP-NWU/log(time+1) was not higher than that of IP-NWU, according to the ROC curves shown in Figure 4B (DeLong test, *p* > 0.05). MCC was 0.49 and 0.54 for IP-NWU/time and IP-NWU/log(time+1), respectively. Broocks et al. reported a similar result (16).

### Image Patches vs. Segmented Regions

**Figure 5:**
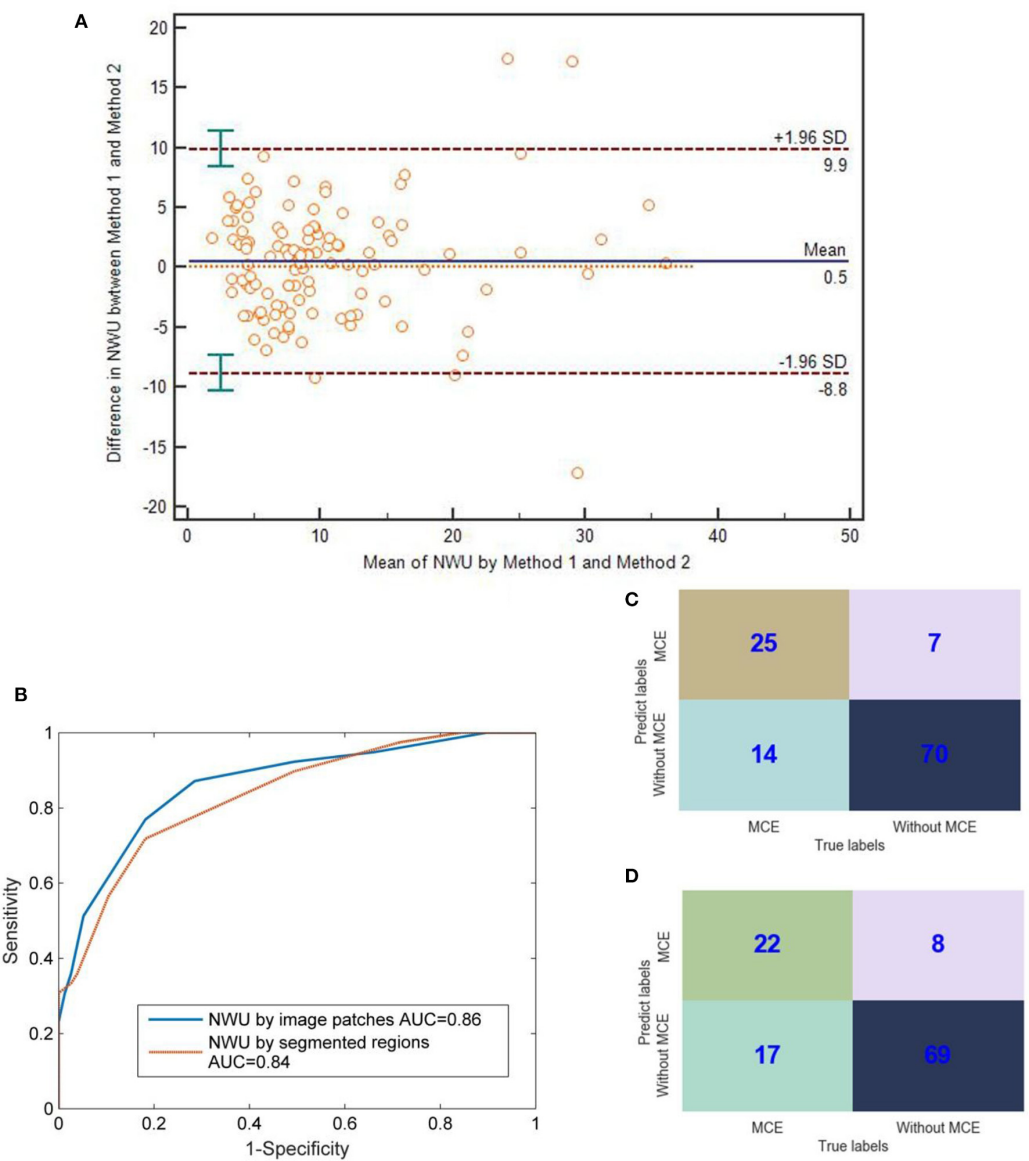
Comparison between IP-NWU and SR-NWU and their predictive performances for malignant cerebral edema (MCE). **(A)** Consistence of IP-NWU and SR-NWU; **(B)** ROC curves of MCE prediction by IP-NWU and SR-NWU; **(C)** The confusion matrix of MCE prediction by IP-NWU; **(D)** The confusion matrix of MCE prediction by SR-NWU.

As for the value of NWU, there was no significant difference between the methods of image patches and segmented regions (Bland-Altman test, *p* > 0.05; Figure 5A). Most points of difference (113 of 116) were located within the 95% limits of agreement (1.96 standard deviation).

In terms of the prediction of MCE, as shown in Figure 5B, IP-NWU had better performance than SR-NWU (AUC: 0.85 vs. 0.83). However, no significant difference was observed (DeLong test, *p* > 0.05; MCC, 0.58 vs. 0.55). Using the comparison of confusion matrices, one also can find that IP-NWU had higher SEN (0.64 vs. 0.56), SPE (0.91 vs. 0.90), and ACC (0.82 vs. 0.78) than SR-NWU (Figures 5C,D).

### Inter-reader and Interoperation Agreement of IP-NWU

**Figure 6:**
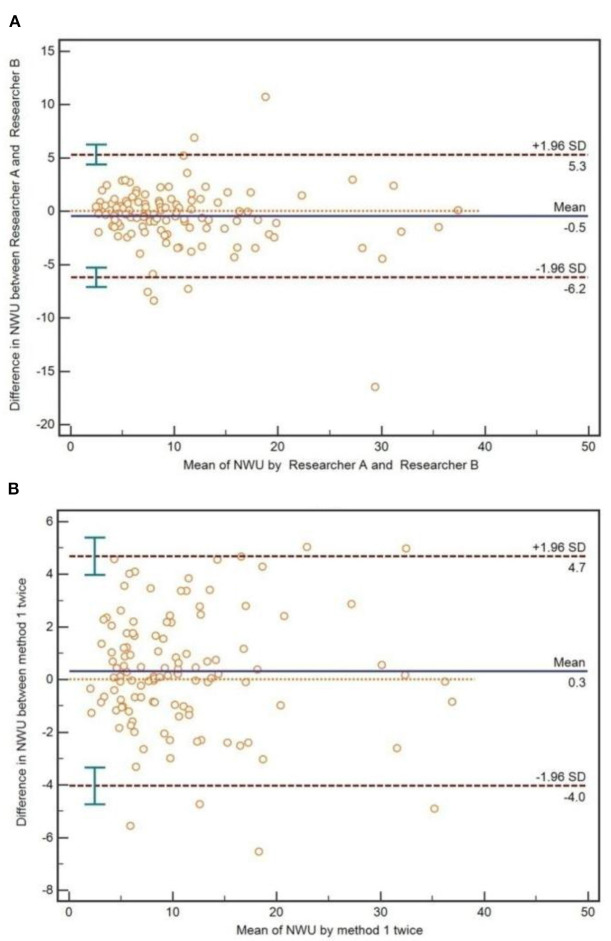
Inter-reader and interoperation agreement for image patch-based net water uptake (IP-NWU). **(A)** The inter-reader agreement, evaluated by the Bland-Altman method; **(B)** The interoperation agreement, evaluated by the Bland-Altman method.

As for the method of IP-NWU, there was an exceptional inter-reader agreement (ICC is 0.92). The Bland-Altman method also indicated that there was no significant difference between Researcher A and Researcher B regarding the measurement of IP-NWU (*p* > 0.05; Figure 6A). Most points were located within the 95% limits of agreement. Meanwhile, as shown in Figure 6B, no significant difference was observed between the two measurements by the same reader (*p* > 0.05), indicating good reproducibility of the image patch method. The ICC of the two measurements was 0.95.

### Radiomics Model for Predicting MCE

As shown in Table 2, for all three machine learning algorithms, SVM, LR, and RF adding the clinical information of age, gender, and NIHSS scores of the patients onto IP-NWU did not improve prediction of MCE. For SVM and LR, compared with the prediction when only using IP-NWU, neither features of “NWU + Imaging” nor “NWU + Clinical + Imaging” improved the performance of predicting MCE.

**Table 2:** Performance comparison of different radiomics models for predicting MCE.

**Classifier**	**Features**	**ACC**	**SEN**	**SPE**	**AUC**	**F1-score**	**PPV**	**NPV**
SVM	NWU + Clinical	0.80	0.62	0.88	0.85	0.72	0.72	0.82
	NWU + Imaging	0.84	0.64	0.94	0.84	0.85	0.83	0.84
	NWU + Clinical + Imaging	0.83	0.62	0.94	0.84	0.80	0.83	0.83
LR	NWU + Clinical	0.80	0.59	0.91	0.85	0.72	0.77	0.81
	NWU + Imaging	0.80	0.59	0.91	0.83	0.72	0.77	0.81
	NWU + Clinical + Imaging	0.81	0.62	0.91	0.81	0.73	0.78	0.82
RF	NWU + Clinical	0.77	0.62	0.84	0.83	0.74	0.67	0.81
	NWU + Imaging	0.91	0.85	0.94	0.96	0.90	0.87	0.92
	NWU + Clinical + Imaging	0.91	0.85	0.95	0.96	0.91	0.89	0.92

**Figure 7:**
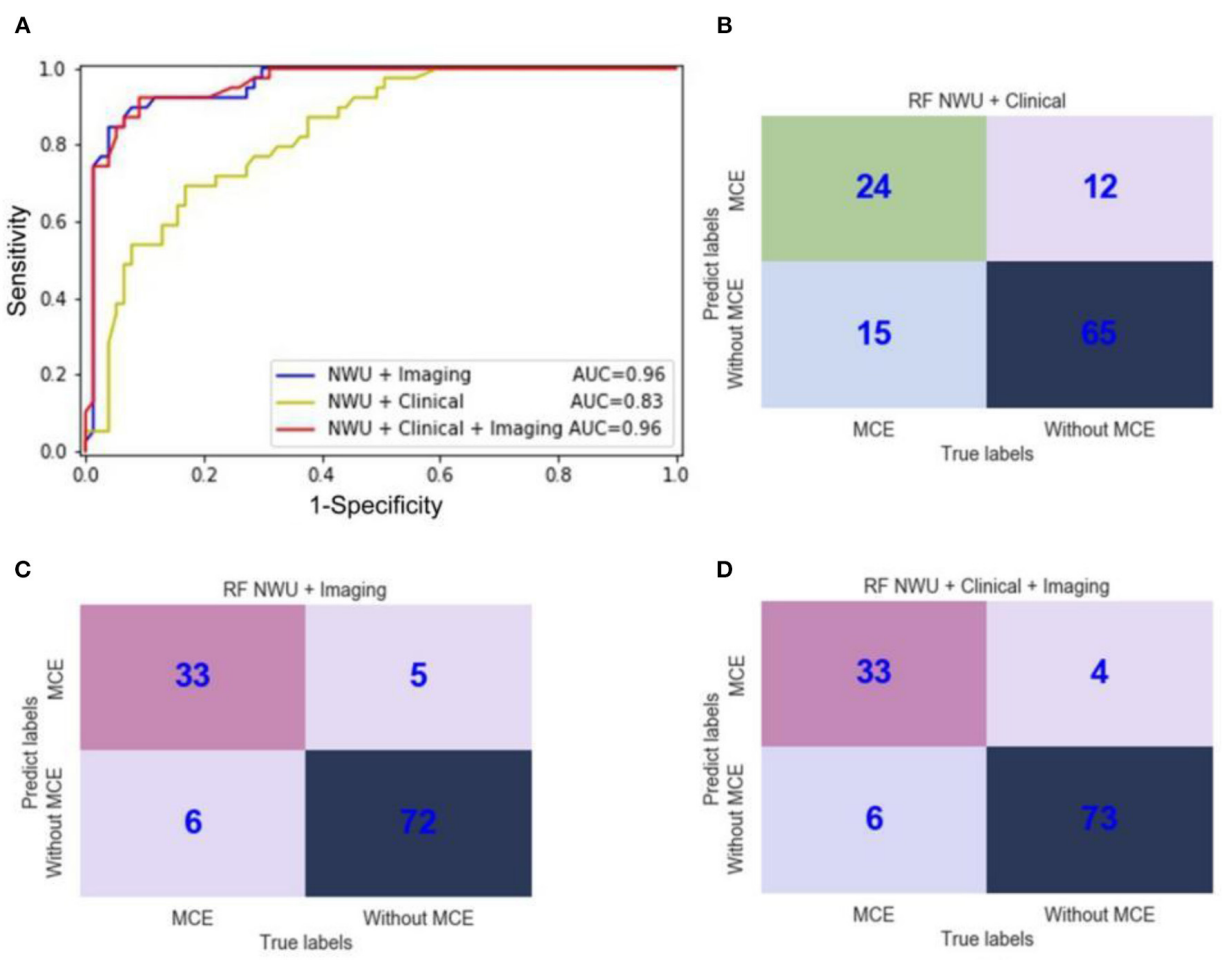
ROC curves and confusion matrices of three random forest (RF) radiomics models for the prediction of malignant cerebral edema (MCE). **(A)** ROC curves of three RF radiomics models with different features; **(B)** The confusion matrix for a model with RF and features of “NWU + Clinical;” **(C)** The confusion matrix for a model with RF and features of “NWU + Imaging;” **(D)** The confusion matrix for a model with RF and features of “NWU + Clinical + Imaging”.

However, as for RF, the features of “NWU + Imaging” can significantly increase the ACC to 0.91, SEN to 0.85, SPE to 0.94, AUC to 0.96, F1-score to 0.90, PPV to 0.87, NPV to 0.92, and MCC to 0.79 (Table 2, Figure 7; DeLong test, *p* < 0.05). When adding the clinical information, no significant improvement was observed (DeLong test, *p* > 0.05; MCC, 0.80 vs. 0.79). This means that the performance of the radiomics model for predicting MCE depends on both the classifiers and features. In the current study the combination of RF and features of “NWU + Clinical + Imaging” had the best performance. For this model, 73 of 77 patients who did not develop MCE were accurately predicted and 33 of 39 patients who developed MCE were accurately predicted.

## Discussion

The aim of this study was to calculate net water uptake (NWU) using admission non-enhanced CT image patches scanned within 24 h from stroke onset and build predictive models for MCE by combining NWU with other features using a radiomics approach. The main findings had four aspects: (1) NWU can be estimated by using the standard reference images and patches; (2) the results for IP-NWU showed no significant difference with results when using segmented regions; (3) IP-NWU is a predictor of MCE; and, (4) radiomics models using IP-NWU and other imaging features can predict MCE rather precisely.

### Standard Reference Images and Patches: An Exceptional Method for Calculating Net Water Uptake

Net water uptake in the ischemic regions was originally proposed to identify patients with stroke onset within 4.5 h (the time window of thrombolysis) and extended to predict malignant infarction in 2018 (15, 16). It relies on the high sensitivity of CT perfusion to precisely locate the infarct core and the high specificity of non-enhanced CT to measure density. However, for many stroke centers and patients, the CTP and its postprocessing for quantitative perfusion maps, including cerebral blood volume, cerebral blood flow (CBF), mean transit time (MTT), and time to drain (TTD), are not accessible.

The early hypoattenuation of the ischemic core in non-enhanced CT images is uncertain or difficult to detect. However, the anatomic location of the MCA (the potential target of infarction) is known. Therefore, we proposed a way of calculating NWU using the standard reference images and patches. Our results showed that this method enables presenting a significant difference in NWU between patients with MCE and without MCE. Further study of the inter-reader agreement in NWU calculation demonstrated that the method had good reproducibility. In summary, using the standard reference images and patches is an exceptional way of calculating net water uptake. It is easy to implement, reliable, and does not require the aid of CTP, CT angiography, or manual segmentation.

Recently, NWU has been used to quantify the treatment effects for ischemic stroke; e.g., thrombectomy and adjuvant drugs, especially for the cases with uncertain indications for treatment, such as low ASPECTS (35). Therefore, IP-NWU may be extended to similar applications.

### Reference Images and Patches vs. Segmented Ischemic Regions

Locating the infarct core by overlaying segmented ischemic regions from follow-up CT images (>24 h after stroke onset) is an alternative method compared to the standard method of using a CBV map from CTP. No significant difference was observed between IP-NWU and SR-NWU, which supports the evidence that IP-NWU is accurate.

However, it is still not clear to what extent the follow-up CT images can work as surrogates of CTP. The ischemic core, penumbra, and benign oligemia cannot be differentiated from the follow-up CT images (36). The registration error between Image-A and Image-F may have a further detrimental effect on NWU calculations.

### Predictor of MCE and Other Confounders

IP-NWU can be a predictor of MCE for middle cerebral artery stroke patients with an AUC of 0.85. Moreover, the prediction was not influenced by interventional thrombectomy, which is in agreement with a previous study (16). The decision to take IT is based on an early infarct and hypoattenuation. Specifically, patients with a large volume of early infarct and visually evident areas of hypoattenuation are potentially excluded from IT. However, the recanalization status and its influence on IP-NWU and MCE are unknown and need further investigation (37, 38). Complete recanalization does not directly indicate a good clinical outcome (39). It has been noted that the prediction of MCE is different from that of cerebral edema (40, 41). Moreover, the rate of IT in our current study (15.52%, 18 of 116) might be lower than that in developed countries due to the high economic cost and late presentations at the hospital.

The relationship between IP-NWU and time in patients with MCE and without MCE is also in accord with that reported in a previous study (16); i.e., NWU increases with time from stroke onset. However, there was no significant difference in the AUC for MCE prediction among NWU, NWU/time, and NWU/log(time+1).

### Radiomics Leverages Machine Learning and Features to Predict MCE Precisely

Our radiomics model using RF and features of “NWU+Clinical+Imaging” had a comparable performance with the model reported by Broocks et al. (AUC: 0.96 vs. 0.93) (16). This indicated that using more imaging features might be superior to only using NWU. Moreover, the possible reason may rely on two aspects: (1) CTP was not used to locate the ischemic core; and (2) our data consisted of 33.6% (39 of 116) of patients with MCE, which was higher than that in a study by Broocks et al. (18.2%) (16). Our model also showed a higher AUC than that of EDEMA scores (AUC = 0.72) (10) and that of modified EDEMA scores by adding NIHSS scores for 478 Chinese patients (AUC = 0.80) (11).

Radiomics leverages machine learning and quantitative imaging features to improve the prediction of clinical outcomes (18). For the prediction of MCE in our current study, radiomics worked well. By adding 13 imaging features from histogram analysis of voxel-wised IP-NWU maps, the AUC of the classifier by RF can increase from 0.86 to 0.96. This improvement may be due to the fact that: (1) multivariate analysis by machine learning has more predictive power than the univariate ROC analysis; and, (2) IP-NWU is only the mean of IP-NWU maps, and more measures from these maps represent the characteristics of the core lesions better. The lesion volume, texture features, penumbra pattern, and other high-level abstract features may be helpful and should be included in radiomics models of MCE predictions in the near future.

All 13 imaging features extracted from histogram analysis were used without selection in our current study. Unlike the application of the PyRadiomics Python package in tumor imaging, more than 1,000 features are extracted (https://pyradiomics.readthedocs.io/). Hence feature selection must be done to reduce overfitting. During feature selection, the importance of features can be obtained (42). Since we only used 13 imaging features, feature selection, and importance analysis were not undertaken in this study. The reason why the PyRadiomics Python package was not used to extract more than 1,000 features is that the voxel-wised IP-NWU map has a small size of 30 × 30 voxels. This map does not contain rich information such as the locations of tumor lesions.

For the predictive radiomics model, there is a danger of overfitting if the number of features is very large. In our study, the largest number of features was 19 (IP-NWU, 13 imaging features, and 3 clinical features) and 116 samples or patients were included. According to a rule of thumb in radiomics, each feature requires 10 samples (18). Therefore, the overfitting in our study should be minimal.

In the present study, RF performed better for MCE prediction than SVM and LR. For example, the model using RF and “NWU + Clinical + Imaging” had an AUC of 0.96, while the model using LR and SVM had an AUC of 0.81 and 0.84, respectively. RF usually performs best in situations where the output is highly sensitive to small changes in input (29). This may indicate that the prediction of MCE is highly sensitive to small changes in NWU and imaging features. Moreover, RF is one ensemble or consensus estimator, and thus has the merit of mitigating both underfitting and overfitting (29). Underfitting and overfitting may exist in the current study given the fact that the sample size was small.

Adding the clinical information of age, gender, and NIHSS scores did not significantly improve the prediction of MCE, which agreed with another previous report (16).

### Limitations and Future Works

Our study has some limitations that could provide direction to future studies. First, our study was retrospective and limited to one single center. The relevance of the resulting radiomics models is unknown for data from other hospitals. Second, the number of patients (116) was relatively small, which limits the statistical power of the study. Third, the selection of slices and patches was done by experts, making IP-NWU depend on expert conditions, such as preferences, experiences, and mood. To set image patches in the cortex orientated by ASPECTS regions may further improve the presented method. It is noted that the cortex regions with CSF should be avoided to eliminate the effect of CSF on NWU. This criterion may make some early infarcts in the cortex not represented in the image patches. Finally, we used three machine learning algorithms and manually designed histogram-based imaging features.

A prospective and multi-center study with CTP and non-enhanced CT scans should be carried out in the near future, before the proposed IP-NWU and radiomics models are introduced as clinical applications. The automatic and machine learning based detection of early infarctions from non–contrast-enhanced CT images should be used to help calculate NWU and predict MCE (43, 44). Other features such as texture and high-level abstract representation can be included. As a state-of-the-art example of deep learning, a deep convolutional neural network (DCNN) may help predict MCE directly according to the image patches or infarction regions (45–47). Given the fact that a stroke is a dynamic process, using both admission and follow-up CT images to characterize the temporal and spatial development of infarcts and edema volume may further improve the final prediction of clinical outcomes.

## Conclusion

Net water uptake can be calculated based on mirrored patches that are selected by senior neuroradiologists from admission non-enhanced CT images that were scanned within 24 h after stroke onset using standard reference images. The resulting IP-NWU values showed a significant difference between patients with MCE and without MCE and thus it is an effective predictor of MCE. The inter-reader and interoperation agreement for IP-NWU are exceptional. Through integrating IP-NWU and other imaging features by machine learning, the radiomics models further improved the prediction of MCE. In summary, this study demonstrated the feasibility of predicting MCE using only admission non-enhanced CT images scanned within 24 h after onset, even without the aid of CT perfusion or follow-up CT scans. This will potentially help clinicians make decisions about performing a surgical decompressive craniectomy or employing other intensive monitoring to benefit stroke patients.

## Data Availability Statement

The original contributions presented in the study are included in the article/supplementary materials, further inquiries can be directed to the corresponding author.

## Ethics Statement

The studies involving human participants were reviewed and approved by the Medical Ethics Committee of General Hospital of Northern Theater Command. Written informed consent for participation was not required for this study in accordance with the national legislation and the institutional requirements.

## Author Contributions

SQ, YK, YY, and HC designed and directed the study. BF, SQ, LT, HX, BY, and YD analyzed data. LT, HX, and HC recruited participants and acquired data. BY and YD reviewed the CT images and drew the image patches. BF, SQ, YK, and YY drafted the manuscript together. All authors revised and approved the final manuscript.

## Conflict of Interest

The authors declare that the research was conducted in the absence of any commercial or financial relationships that could be construed as a potential conflict of interest.
